# Delayed posthypoxic leukoencephalopathy: a case series and review of the literature

**DOI:** 10.1002/brb3.364

**Published:** 2015-07-03

**Authors:** Carlos A Zamora, David Nauen, Robert Hynecek, Ahmet T Ilica, Izlem Izbudak, Haris I Sair, Sachin K Gujar, Jay J Pillai

**Affiliations:** 1 Division of Neuroradiology, The Russell H. Morgan Department of Radiology and Radiological Science, Johns Hopkins University School of Medicine601 N Caroline St, Baltimore, MD, 21287; 2 Department of Pathology, Johns Hopkins University School of Medicine600 N Caroline St, Baltimore, MD, 21287

**Keywords:** Hypoxia, leukoencephalopathy, MRI, myelin, white matter

## Abstract

**Background:**

Delayed posthypoxic leukoencephalopathy (DPHL) is a rare and underrecognized entity where patients manifest a neurological relapse after initial recovery from an acute hypoxic episode. We sought to describe the magnetic resonance imaging (MRI) findings in a group of patients with DPHL and review the available literature.

**Methods:**

Retrospective case series including patients who presented with neurological and/or psychiatric symptoms after recovery from an acute hypoxic episode. The history and clinical presentation were reviewed from the electronic medical records. MRI scans were evaluated from the picture archiving and communication system. We performed a comprehensive review of the English medical literature for prior published cases of DPHL and describe the key imaging findings that have been reported related to this condition.

**Results:**

A total of five patients were identified, including four patients with respiratory failure due to drug overdoses from benzodiazepines, opioids, and/or barbiturates, and one patient who presented after cardiopulmonary arrest due to pulmonary embolism. All patients showed diffuse, extensive, and confluent white matter signal abnormalities including prominent restricted diffusion, extending to the subcortical white matter and respecting the U-fibers. There was no gyral edema or contrast enhancement. In one case histopathology was available, which highlighted patchy subcortical myelin loss with sparing of U-fibers and demonstrated prominent macrophage/microglial inflammation with extensive axonal damage. Of the other four patients, two were at their neurological baselines and two had persistent neurological deficits at the time of discharge.

**Conclusions:**

The described constellation of MRI findings is highly suggestive of DPHL in the appropriate clinical setting.

## Introduction

Delayed posthypoxic leukoencephalopathy (DPHL) is a rare and underrecognized entity characterized by neurological relapse following a period of clinical stability or improvement after an episode of hypoxia. Patients usually present between one and 4 weeks after the initial event with relatively lucid intervening periods of variable lengths. While the exact mechanism behind its delayed manifestation has not been elucidated, DPHL is considered a distinct process from various direct causes of acute leukoencephalopathy such as toxic and metabolic injury. The majority of DPHL cases reported to date have been associated with carbon monoxide intoxication, where the incidence is approximately 2.8% (Choi 1983). However, delayed neurological sequelae have also been documented in other causes of hypoxia, particularly due to respiratory failure in drug overdoses (Rozen 2012; Salazar and Dubow 2012; Meyer 2013).

Histopathologically, DPHL is characterized by widespread demyelination with axonal preservation (Gottfried et al. 1997). Diffuse and confluent white matter changes are present on magnetic resonance imaging (MRI), most notably with extensive, symmetric, and often striking restricted diffusion (Molloy et al. 2006). Such a pattern is distinctly different from that seen in acute hypoxic-ischemic injury in the adult, which involves predominantly the gray matter structures (Huang and Castillo 2008). Although DPHL as a clinical phenomenon has been known for many years, the presence of extensive restricted diffusion has only been recognized after the relatively recent advent of diffusion-weighted imaging (DWI) and probably constitutes the most remarkable MRI feature. To date, the literature regarding MRI findings is relatively scarce and consists of scattered case reports on patients with various etiologies of hypoxia and two small series of DPHL after carbon monoxide poisoning (Kim et al. 2003; Hsiao et al. 2004). Herein, we describe the MRI characteristics of DPHL in a series of five adult patients, the majority of whom presented following drug overdoses, with histopathologic correlation in one case. We also offer an extensive review of the literature including cases where MRI was performed.

## Materials and Methods

### Case series

This retrospective case series was approved by our institutional review board which waived requirement for informed consent. Adult patients (≥18 years of age) who fulfilled the following criteria were included in the study: (1) neurological deterioration caused by an initial hypoxic event; (2) subsequent clinical improvement with return to (prehypoxic event) baseline or near baseline; and (3) neurological relapse or new neurological or psychiatric symptoms following clinical improvement. Patients with continued deterioration after the initial hypoxic event without a lucid period or a clear relapse or with an alternative explanation for neurologic deterioration were excluded.

### Imaging technique

Studies were performed on 1.5 T (Magnetom Aera, Siemens, Erlangen, Germany; Signa, GE Healthcare, Milwaukee, WI) or 3.0 T (Magnetom Skyra, Siemens) MRI scanners and typically consisted of T1- and T2-weighted sequences, T2 fluid-attenuated inversion recovery (FLAIR), trace isotropic DWI at *b* = 1000, and gadolinium-enhanced T1-weighted sequences. Parameters were as follows: for T2 FLAIR, TR = 8000–9000, TE = 101–126, and IR = 2200–2500; for T2, TR = 2683–5940, and TE = 102–117; and for DWI, TR = 7500–10,000, TE = 80–99, number of excitations = 1 or 2, matrix = 128 × 128 to 192 × 192; field of view, 22 × 22 cm to 24 × 24 cm, slice thickness = 4–5 mm, and gap 4–6.5 mm.

### Data collection and analysis

The electronic medical records of each patient were reviewed to determine the clinical presentation, history, etiology of hypoxia, time to neurological relapse, and clinical manifestations during relapse. MRI studies were extracted from our picture archiving and communication system. Time from the initial hypoxic event to identification of diffuse white matter abnormalities on MRI (FLAIR and DWI) was recorded. The following imaging characteristics were visually analyzed: morphology (patchy or homogeneous), symmetry, relative extent of FLAIR and ADC abnormalities, spared structures, presence of mass effect or gyral edema, contrast enhancement, or hemorrhage.

### Histopathology

For the autopsy case, the brain was fixed in formalin for 2 weeks. Following brain cutting representative sections were taken for microscopic assessment. Histochemical staining was done with hematoxylin and eosin, with Luxol fast blue added to label myelin. Immunohistochemistry was performed to detect expression of SM31 (Sternberger) for neurofilaments and CD68 (Ventana) for macrophages.

### Literature review

We queried the PubMed database using the following terms for articles written in the English language: *“delayed hypoxic encephalopathy”*, *“delayed hypoxic leukoencephalopathy”*, *“delayed leukoencephalopathy”*, and *“delayed hypoxic-ischemic leukoencephalopathy”*, as well as various permutations substituting *“reversible”* for *“delayed”* and *“post-hypoxic”* for *“hypoxic”* and *“post-anoxic.”* All articles were reviewed for redundancy of patients and only those who had a clear delayed presentation fulfilling the criteria above and who underwent MRI were included.

## Results

### Patient characteristics

A total of five patients (three men and two women) fulfilled criteria for inclusion in this case series. Median age (interquartile range) at presentation was 63 years (59–64). All patients were 59 years or older except for one patient who was 32 years old.

### Clinical presentation and course

Four patients were brought to our facility following an episode of respiratory failure due to drug overdose with opioids, benzodiazepines, and/or barbiturates. Three of them were found unresponsive and one had altered mental status in the setting of respiratory failure. One of five patients developed pulmonary embolism at home 8 days after being discharged for bowel surgery and was brought to the hospital in cardiorespiratory arrest. After this initial admission for respiratory failure/hypoxia, all patients were discharged from the hospital at their baseline or near baseline. Relapse was made manifest with neuropsychiatric symptoms such as erratic behavior, ataxia, urinary and/or fecal incontinence, delusions, akinetic mutism, and deficits in executive functioning including memory and attention. Two patients presented with pyramidal signs at relapse, consisting of triple flexion response on plantar stimulation (patient 2) and a pronator drift (patient 3). Other symptoms included increased tone, tremors, and cogwheel and leadpipe rigidity. Median time to relapse was 23 days (14–32). One patient continued to worsen after relapse and died 24 days after readmission. The other four patients progressively improved and were discharged at a median 24.5 days (21.5–33.75) after relapse. Two out of four patients were at their neurological baselines at discharge. The other two patients had shown significant improvement but had persistent deficits (Table 2000).

**Table 1:** Clinical characteristics of patients with delayed posthypoxic leukoencephalopathy

Patient No./age (years)/sex	Etiology of hypoxia	Initial manifestations	Time to relapse	Manifestations during relapse	Condition at discharge
1/64/M	Cardiopulmonary arrest due to pulmonary embolism	Unresponsive	23 days	Progressive cognitive deterioration, psychomotor retardation, and global weakness; increased tone, cogwheeling, tremor	Alert and oriented but with attention and memory deficits and failure to follow multi-step commands
2/32/M	Opioid overdose	Respiratory distress and altered mental status	32 days	Bizarre behavior, urinary and fecal incontinence, akinetic mutism; tremor, triple flexion response on plantar stimulation	Alert and oriented, at neurological baseline
3/63/F	Opioid, benzodiazepine, and barbiturate overdose	Unresponsive	5 weeks	Delusions, memory deficits, decreased mood, and akinetic mutism. Postural tremor, pronator drift	Alert and oriented, at neurological baseline
4/65/M	Opioid and benzodiazepine overdose	Unresponsive	2 weeks	Odd behavior including disinhibition and paranoia; increased tone, slow and shuffling gait	Much improved but persistent unsteady gait; deficits of executive functioning
5/59/F	Oxycodone overdose	Unresponsive	2 weeks	Irrational behavior, mania, parkinsonism, and catatonia. Leadpipe rigidity	Deceased

### MRI findings

**Figure 1:**
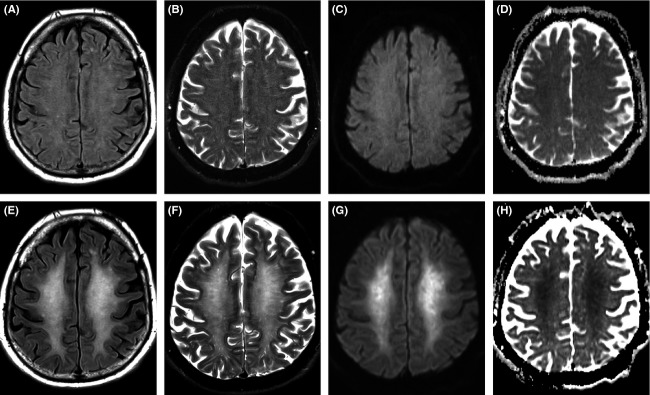
65-year-old male presenting after an acute hypoxic episode secondary to opioid and benzodiazepine overdose (case 4). Baseline MRI (A through D) demonstrate a few nonspecific subcortical T2 and FLAIR white matter hyperintensities (A and B) without restricted diffusion (C and D) probably representing chronic small vessel ischemic changes in a patient of this age. MRI after neurological relapse (E through H) shows diffuse confluent white matter abnormalities in the centrum semiovale involving the subcortical white matter. There is corresponding signal hyperintensity on DWI (G) with somewhat less extensive hypointensity on the calculated ADC maps (H), indicating some degree of T2-shine through superimposed on cytotoxic edema. FLAIR: TR = 9000, TE = 126, and IR = 2490; DWI: TR = 9000, TE = 98, number of excitations = 2, matrix = 192 × 192; field of view, 23 × 23 cm to 24 × 24 cm, slice thickness = 4 mm, and gap 4.

**Figure 2:**
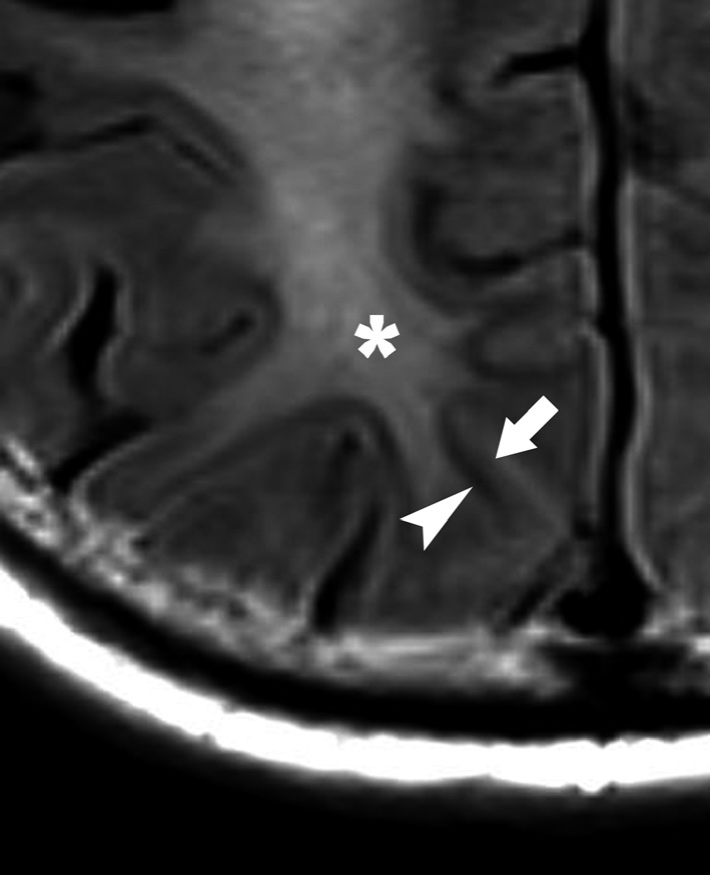
Cropped and digitally magnified region from Fig.1E demonstrates that the white matter signal abnormality (asterisk) involves the subcortical white matter but spares the U-fibers which appear as a curvilinear dark band (arrowhead). The adjacent gray matter is also visualized (arrow).

Median time to identification of white matter abnormalities since the initial hypoxic event was 40 days (30–50). All cases demonstrated extensive and confluent T2 and FLAIR hyperintensity involving predominantly the periventricular white matter and centrum semiovale, bilaterally and in a symmetric fashion (Fig.1). In two out of the five patients, restricted diffusion matched the extent of the T2-FLAIR hyperintensity, while in three patients the restrictive abnormalities were relatively less extensive. The white matter lesions were confluent and homogeneous in two patients. Three patients had evidence of more heterogeneous, patchy lesions which still followed an overall symmetric distribution. In all patients the T2 abnormalities involved the subcortical white matter but spared the U-fibers (Fig.2), and were confined to the supratentorial brain without affecting the basal ganglia, thalami, brain stem, or cerebellum. There was no gyral edema or sulcal effacement, and there were no areas of contrast enhancement following the intravenous administration of gadolinium (Table 2004). These findings were new in four out of the five patients in whom baseline MRI studies from their initial presentation were available for review. In one out of five patients, we did not have the initial imaging studies from an outside institution; however, these reportedly did not show an acute abnormality. Initial baseline MRI on patient number four showed three small subacute-appearing embolic infarcts in the supratentorial brain, without the confluent and symmetric white matter abnormalities that were seen on an MRI study performed 9 days later. Of the other patients who had a baseline study, two demonstrated chronic white matter ischemic changes and one did not show any significant findings.

**Table 2:** MRI characteristics in a series of patients with delayed posthypoxic leukoencephalopathy

No.	Time to MRI^1^ (days)	Morphology of signal abnormality	Symmetry	T2-FLAIR/ADC mismatch	Spared structures	Mass effect or gyral edema	Contrast enhancement	Hemorrhage
1	40	Patchy	Yes	T2-FLAIR more extensive than ADC	U-fibers, brainstem, basal ganglia, thalami, cerebellum	No	No	No
2	50	Homogeneous	Yes	T2-FLAIR more extensive than ADC	Same as above	No	No	No
3	54	Patchy	Yes	Matched T2-FLAIR/ADC	Same as above	No	No	No
4	30	Homogeneous	Yes	T2-FLAIR more extensive than ADC	Same as above	No	No	No
5	20	Patchy	Yes	Matched T2-FLAIR/ADC ADC	Same as above	No	No	No

1 Time to MRI abnormality since the original hypoxic event (during relapse).

MRI, magnetic resonance imaging; ADC, apparent diffusion coefficient; FLAIR, fluid-attenuated inversion recovery.

### Histopathologic findings

**Figure 3:**
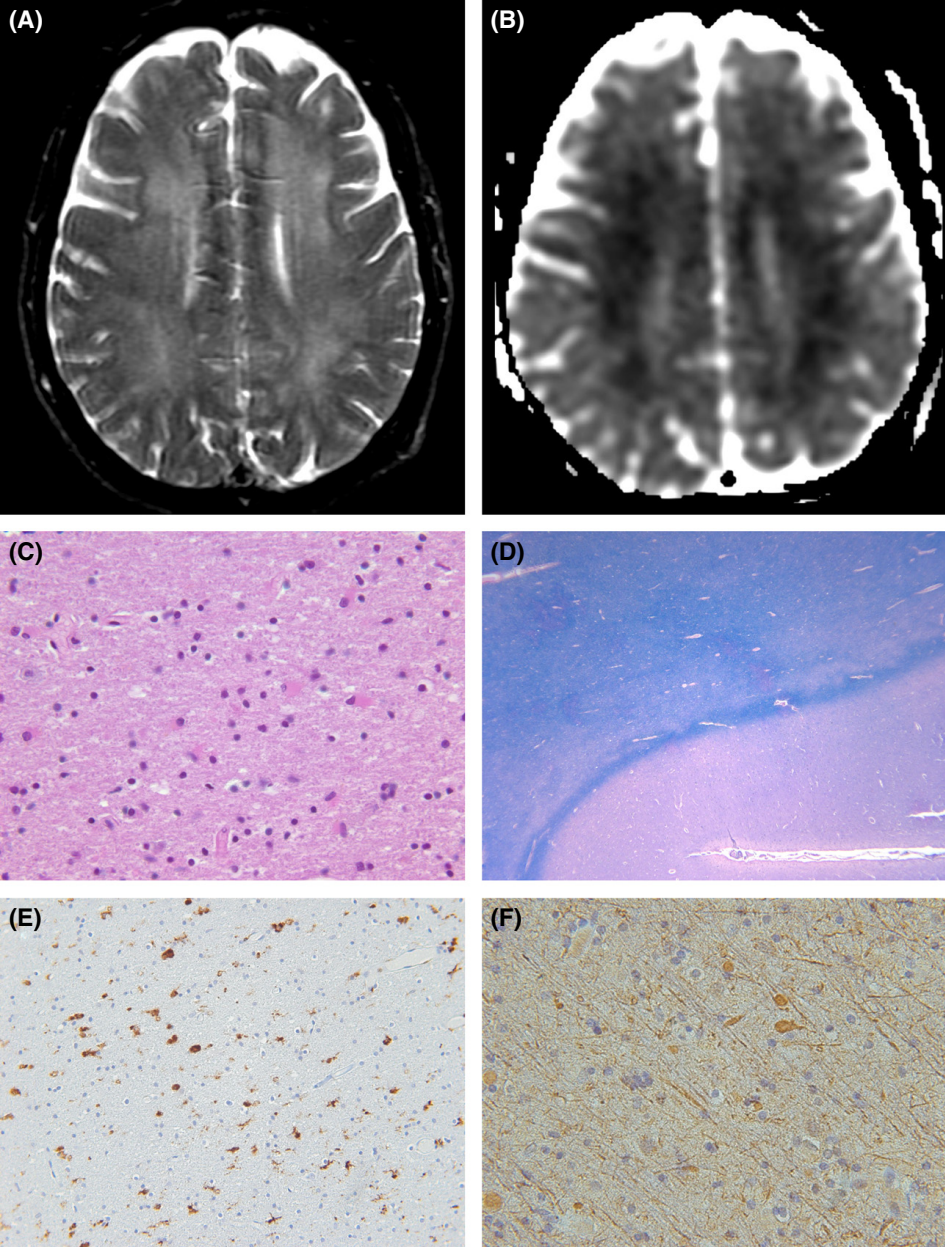
MRI and histologic sections from a 59-year-old female who developed DPHL after being found unresponsive in the setting of opioid overdose. Axial T2-weighted image demonstrates more patchy white matter lesions compared to the case in Fig1, with corresponding low apparent diffusion coefficient (B) indicating restricted diffusion. T2: TR = 2683 and TE = 103; DWI: TR = 10,000, TE = 80, number of excitations = 2, matrix = 128 × 128, field of view, 24 × 24 cm, slice thickness = 5 mm, and gap 5 mm. (C) Abundant reactive astroglia characterized by brightly eosinophilic cytoplasm and enlarged nuclei are numerous in subcortical white matter. The mildly vacuolated background neuropil demonstrates ubiquitous eosinophilic ‘blebs’ which impart a rough or stippled character. Occasional macrophages are appreciated, for example, at 9 o’clock position. Hematoxylin and eosin, original magnification 200×. (D) The subcortical u-fibers at the gray white junction exhibit normal myelin, but white matter beneath these shows marked patchy pallor. Hematoxylin and eosin with luxol fast blue, original magnification 20×. (E) CD68 stain highlights lysosome-enriched cells, microglia and macrophages, abundant in the neuropil. Immunohistochemistry for CD68, original magnification 100×. (F) Neurofilament stain highlights frequent axonal swelling, indicative of damage, in white matter. Immunohistochemistry for SM31, original magnification 200×.

Autopsy examination of the brain in case 5 demonstrated no significant edema or sulcal effacement. Microscopically, there was extensive white matter injury with myelin loss and axonal swelling, as well as abundant reactive astroglia, in a mildly vacuolated background neuropil. These changes involved the subcortical white matter in a patchy distribution, although the U-fibers were preserved (Fig.3).

## Discussion

The gray matter of the adult brain demonstrates selective vulnerability to acute hypoxic-ischemic injury, which is triggered by a complex cascade of cellular events resulting in glutamate excitotoxicity, release of free radicals, and apoptosis (Won et al. 2002). Hypoxia preferentially affects the basal ganglia, thalami, neocortex, and hippocampus, with relative sparing of the brainstem, and results in a consistent pattern of abnormalities on MRI which are most evident on DWI within the first few hours following injury (Huang and Castillo 2008). On the contrary, white matter is relatively resilient to the effects of hypoxia, with the exception of carbon monoxide poisoning, which can cause acute demyelination preferentially damaging the globi pallidi and subcortical white matter (Adams et al. 2000).

In the setting of drug overdose, there are two principal mechanisms by which white matter can be injured. First, a form of spongiform leukoencephalopathy with intramyelinic vacuolization has been described from inhalation of the pyrolysate vapors produced after heating heroin in a practice known as “chasing the dragon”, which primarily presents as a cerebellar syndrome causing ataxia, dysarthria, and bradykinesia (Won et al. 2002; Keogh et al. 2003). White matter injury in heroin inhalation encephalopathy favors the cerebellum, brainstem, posterior cerebral white matter, and posterior limb of the internal capsule (Keogh et al. 2003). This is in contradistinction to a second mechanism consisting of delayed demyelination, seen in DPHL, which occurs weeks after the initial hypoxic event and typically spares the cerebellum, brainstem, and basal ganglia. This particular distribution was evident in our study and is also concordant with the MRI findings of DPHL in the majority of published case reports and series. Findings reported in the literature are described in detail in Table 2012 (Weinberger et al. 1994; Lee and Lyketsos 2001; Arciniegas et al. 2004; Molloy et al. 2006; Shprecher et al. 2008; Mittal et al. 2010; Wallace et al. 2010; Nzwalo et al. 2011; Huisa et al. 2013; Meyer 2013; Tormoehlen 2013; Geraldo et al. 2014).

**Table 3:** Review of studies in the literature including MRI

Year	Author(S)	*N*=	Age	DWI	Follow-up MRI	Etiology of hypoxia	Time to relapse after hypoxia	Major clinical manifestations during relapse	Return to (near) neuro baseline after relapse	MRI findings
1991	Hori et al. (1991)	1	13	No	Yes	Strangulation	7 days	Apathy, drooling, dysarthria; grimacing, choreoathetosis, spasticity, pseudobulbar paralysis	Minimal dystonia on 3-month follow-up. Nearly fully recovered at 1.5 years	No abnormalities on day 10. New lesions in putamen and caudate nuclei bilaterally on day 23, with cavitation 8 months later
1994	Weinberger et al. (1994)	1	34	No	No	Overdose: benzodiazepines	24 days	Confusion, disorientation, incontinence, hallucinations; shuffling gait, flexor plantar responses, primitive reflexes (frontal release signs), hyperreflexia, clonus	Persistent cognitive deficits	Diffuse increased signal in supratentorial WM
1997	Gottfried et al. (1997)	1	36	No	No	Overdose: opioids	24 days	Withdrawal, forgetfulness, dysautonomia, and confusion. Quadraparesis, myoclonic jerks, clonus, hyperreflexia	Yes, unclear timing	Widespread increased T2 signal changes in supratentorial WM; hyperintense foci in globi pallidi; MRS: reduced NAA, elevated Cho, elevated Lac in WM
2001	Lee and Lyketsos (2001)	1	71	No	No	Overdose: benzodiazepines	14 days	Confusion and disorientation but normal initial neurological exams. Increased tone and shuffling gait over the next 2 days	Return to near baseline, however, with slight wide-based gait 6 months after symptom onset	MRI on admission was normal (day 17 after overdose). MRI 3 weeks after relapse showed diffuse WM “periventricular changes”
2003	Kim et al. (2003)	5	54–71	Yes	1 month (1 case)	Carbon monoxide poisoning	1–4 weeks	Memory loss, confabulation, aphasia, akinetic mutism, incontinence; gait disturbance, bradykinesia	No: four patients “much improved” at 7, 5, 8, 5 months, respectively. One patient with persistent clinical symptoms at 6 month	Diffuse supratentorial T2/FLAIR WM changes with low ADC values; hyperintense foci in globi pallidi in one case; symmetric abnormalities in four of five patients. No change on 1-month follow-up
2004	Arciniegas et al. (2004)	1	29	Yes	No	Overdose: benzodiazepines, opioids	3 weeks	Apathy, memory loss and executive dysfunction. Primitive reflexes	No clear return to near baseline	Diffuse bilateral T2/FLAIR hyperintensity in supratentorial WM; increased DWI signal without mention of ADC
2004	Hsiao et al. (2004)^1^	12	11–79	n/a	Yes, unknown timing	Carbon monoxide poisoning	14–45 days	Cognitive impairment, akinetic mutism; sphincter incontinence, gait ataxia, chorea, dystonia, and parkinsonism	“Greatly improved” cognitive impairment; “some patients” with persistent dystonia	Multiple lesions in subcortical WM and basal ganglia, mostly in the globus pallidus, and to a lesser degree in the putamen, and caudate. “Steady improvement” on follow-up MRI
2006	Molloy et al. (2006)	1	40	Yes	6 months	Overdose: opioids	17 days	Restlessness, echolalia, perseveration	Yes, on 9-month follow-up	Extensive bilateral T2 supraventricular WM hyperintensity with restricted diffusion. Improved but persistent abnormalities at 6 months
2008	Shprecher et al. (2008)	3	39–56	Yes	38 weeks and 31 days (2 cases)	Overdose: benzodiazepine, opioids (also consumed cocaine)	4 weeks, 21 days, and 15 days, respectively	Disorientation, catatonia, memory loss, delusions, attention deficits, incontinence; brisk reflexes, Babinski, unsteady gait, rigidity, hyporeflexia (one patient), clonus, pronator drift	Persistent decline in all three cases.	Diffuse bilateral T2/FLAIR hyperintensity in supratentorial WM; restricted diffusion in two out of three cases (no mention of DWI in the other); sparing of posterior fossa and basal ganglia; MRS decreased NAA, no Lac. Persistent abnormalities at follow-up
2009	Lou et al. (2009)	1	62	Yes	No	Arrest after massive gastrointestinal hemorrhage	2–3 weeks	Akinetic mutism; rigidity, hyperreflexia, cogwheeling	Condition did not improve at 3-month follow-up	Extensive bilateral subcortical WM lesions with restricted diffusion. Lesions in globi pallidi (also present on initial admission) and new lesions in substantia nigra
2010	Wallace et al. (2010)	1	28	No	No	Overdose: benzodiazepine, opioids (also consumed cocaine)	Approx 5 weeks	Confusion, disorientation	Yes, unclear timing.	Periventricular WM changes sparing basal ganglia and gray matter
2010	Mittal et al. (2010)	1	38	Yes	2 months	Overdose: benzodiazepines, opioids	Approx 3 weeks	Memory loss, confusion, executive dysfunction	Yes, unclear timing	Diffuse T2 hyperintensity supratentorial WM; no enhancement, “no foci of restricted diffusion”. Unchanged at 2 months
2011	Nzwalo et al. (2011)	1	55	No	3 months	Overdose: benzodiazepines	3 weeks	Akinetic mutism, decerebration, spastic quadriplegia, generalized hyperreflexia, bilateral Babinsky	Unknown	Extensive T2/FLAIR hyperintensity in supratentorial WM. “Stable” appearance at 3 months
2012	Rozen (2012)	1	59	No	No	Overdose: morphine	3 weeks	Confusion, restlessness; tremor, masked facies, bradykinesia, increased tone, hyperreflexia	“Almost completely back to baseline” with mild memory impairment on 7 month follow-up	T2 hyperintensities periventricular and deep WM; T2 hyperintense foci globi pallidi; no mention of DWI
2012	Salazar and Dubow (2012)	1	54	Yes	No	Overdose: opioids	3 weeks	Confusion, lethargy; rigidity, brisk reflexes, extensor plantar responses	Early recovery upon discharge, no further follow-up	Diffuse, confluent, nonenhancing, symmetric T2/FLAIR WM hyperintensity with restricted diffusion including globi pallidi; sparing brainstem and posterior fossa
2012	Betts et al. (2012)	3	46–59	Yes	6 months (2 patients) and 8 years	Overdose: benzodiazepine, opioids, alcohol	17, 24, and 15 days, respectively	Dysfluent language, slurred speech, agitation, disorientation, memory loss, cognitive slowing. No other details on neurological examination	Yes (one patient). Persistent memory and/or executive function deficits in the other two at 3 and 4 month	Extensive T2 hyperintensity in WM, no enhancement in at least one case (no mention in the other two); restricted diffusion mentioned in two cases; MRS: decreased NAA and increased Cho/Cr ratio. Residual abnormalities on follow-up with globi pallidi lesions in one case
2013	Meyer (2013)	1	43	No	“Within one year”	Overdose: benzodiazepines, opioids	3 weeks	Confusion, disorientation, memory loss, incontinence, lethargy, blunt affect, left side neglect; slow gait	Normal mental status “within 1 year of the event”	Confluent diffuse signal change entire supratentorial WM; “hyperintense on DWI” with no mention of ADC; sparing brainstem and posterior fossa; MRS: elevated Cho and Cr, small Lac peak. Normal follow-up MRI
2013	Choi et al. (2013)	1	37	Yes	No	Spinal cord injury	7 days	Decreased consciousness, dysarthria, akinetic mutism; cogwheel rigidity	Persistent cognitive deficits on 3-month follow-up	T2 hyperintensity in frontotemporal WM, basal ganglia, and cortex
2013	Tormoehlen (2013)	1	46	No	No	Carbon monoxide poisoning	2 weeks	Pseudobulbar affect, confusion, memory loss	Unknown	T2/FLAIR hyperintense lesions in the periventricular WM of both cerebral hemispheres
2013	Huisa et al. (2013)	2	19, 32	Yes	58 days and 112 days	Overdose: opioids	7 days and 15 days, respectively	Coma, spasticity, hyperreflexia in one patient. Confusion, insomnia, hallucinations; hyperreflexia, spasticity, in the other patient. flexor plantar responses in both	Persistent deficits in both patients (20 month follow-up in one patient)	Extensive T2/FLAIR hyperintensity in WM with restricted diffusion at the time of hypoxia in 1 case. Diffuse WM abnormalities in the other case. Normalization of ADC, at follow-up
2014	Geraldo et al. (2014)	1	40	Yes	39 days	Carbon monoxide poisoning	1 month	Mutism, distractibility, reduced attention, bizarre behavior; choreiform movements	Unknown	Confluent, symmetric, T2/FLAIR and proton density abnormalities in WM with restricted diffusion; sparing of gray matter, brainstem, posterior fossa; MRS: decreased NAA, elevated Cho, Lac peak

ADC, apparent diffusion coefficient; Cho, choline; Cr, Creatine; DWI, diffusion-weighted imaging; FLAIR, fluid-attenuated inversion recovery; Lac, lactate; MRS, magnetic resonance spectroscopy; NAA, N-acetylaspartate; WM, white matter; MRI, magnetic resonance imaging; MRS, magnetic resonance spectroscopy.

1 Back issue not available for review.

On histopathologic examination, DPHL demonstrates widespread demyelination with axonal sparing as well as macrophages and reactive astrocytes (Plum et al. 1962; Gottfried et al. 1997). The U-fibers and cerebral cortex are noticeably spared (Plum et al. 1962). As opposed to the spongiform leukoencephalopathy seen in heroin pyrolysate (Wolters et al. 1982; Kriegstein et al. 1999), there is usually no intramyelinic vacuolization in DPHL (Plum et al. 1962; Gottfried et al. 1997).

To date, the pathophysiology of DPHL remains elusive. A proposed mechanism relates to the fact that the turnover rates for some myelin-related proteins range between 19 to 22 days, which is close to the average time for clinical relapse after initial injury (Meyer 2013). This would be consistent with our study, where the median time to neurological relapse was 23 days. However, while this is plausible, it would not explain why DPHL is such an uncommon phenomenon. Additionally, there have been reports of patients with decreased levels of arylsulfatase A, which is deficient in metachromatic leukodystrophy, suggesting that this could represent a predisposing factor (Weinberger et al. 1994; Gottfried et al. 1997). However, levels of this enzyme in other reported cases have been normal (Salazar and Dubow 2012). We did not measure arylsulfatase A in our patients. It is also worth mentioning that DPHL appears to occur after mild-to-moderate episodes of hypoxia, as severe hypoxia would likely result in acute hypoxic-ischemic injury with typical damage to gray matter structures.

In our series, the white matter abnormalities were extensive, bilateral, and symmetric, and invariably involved the subcortical white matter while preserving the U-fibers. In particular, a remarkable imaging finding was the presence of extensive restricted diffusion, which has been partially described in some cases (Kim et al. 2002; Arciniegas et al. 2004; Molloy et al. 2006; Shprecher et al. 2008; Lou et al. 2009; Betts et al. 2012; Salazar and Dubow 2012; Huisa et al. 2013). The majority of reports available, however, do not include DWI sequences (Hori et al. 1991; Weinberger et al. 1994; Gottfried et al. 1997; Lee and Lyketsos 2001; Hsiao et al. 2004; Mittal et al. 2010; Wallace et al. 2010; Nzwalo et al. 2011; Rozen 2012; Choi et al. 2013; Meyer 2013; Tormoehlen 2013; Geraldo et al. 2014), and those that do, lack the level of detail that we include in our series, specifically in terms of spared structures, symmetry, and morphology of the signal abnormalities, and presence of T2-FLAIR/ADC mismatch. We have shown that the FLAIR abnormalities were more extensive than the areas of restricted diffusion in three of our patients, although both showed a similar distribution and were symmetric. These findings were not present on baseline MRI, which was available in 80% of our cases, further substantiating the fact that such changes constitute a delayed manifestation rather than a direct effect of acute injury.

While none of the studies in the literature have reported signal abnormalities in the cerebellum or brainstem, there are a few instances where lesions were present in the basal ganglia, presumably related to the hypoxic injury (Hori et al. 1991; Gottfried et al. 1997; Kim et al. 2002; Hsiao et al. 2004; Lou et al. 2009; Betts et al. 2012; Rozen 2012; Salazar and Dubow 2012; Choi et al. 2013). Specific injury to the globi pallidi has been described in several cases, not only in the setting of carbon monoxide poisoning (Kim et al. 2003; Hsiao et al. 2004), but also following drug overdose and arrest after massive hemorrhage (Gottfried et al. 1997; Lou et al. 2009; Betts et al. 2012; Rozen 2012; Salazar and Dubow 2012). The authors of one case report describe isolated injury to the basal ganglia in a patient with delayed encephalopathy after strangulation (Hori et al. 1991). However, this is the earliest case with MRI and it is possible that subtle abnormalities may have been missed. Additionally, no DWI was available at that time. A delayed-onset dystonia following anoxic injury, without white matter abnormalities and progressive over time, has also been reported in two cases (Kuoppamaki et al. 2002).

The lack of gyral edema in our series also supports a delayed presentation rather than acute toxic or metabolic injury. The absence of contrast enhancement argues against an active inflammatory or demyelinating process, which is possible if myelin injury has already occurred. None of the reviewed publications described contrast enhancement, and if such is present an alternative diagnosis should be sought. Additionally, the clinical presentation in DPHL is different from that of acute hypoxia, with most patients showing bizarre behavior, akinetic mutism, psychomotor retardation, and deficits of executive functioning. The presence of pyramidal signs as well as Parkinsonism and other movement disorders is also relatively common. We believe that, in the appropriate clinical setting, the constellation of MRI findings described above is highly suggestive of DPHL, which usually warrants supportive treatment and bears a relatively good prognosis in most patients. Our findings are similar to those presented by Kim et al. (2003), although that study only included patients with DPHL after carbon monoxide poisoning.

The main limitations of our study are related to the small number of subjects and its retrospective design, which are difficult to avoid given the rarity of this disorder. While a prospective cohort study (e.g., including all patients with neurological deterioration caused by an initial hypoxic event) would be ideal, this type of research is not well suited for rare diseases as an impractically high number of study subjects would be required (Mann 2003). It is possible that we could have identified more cases but the delayed presentation of this entity and the fact that many patients may present primarily with psychiatric symptoms (Tormoehlen 2013) may cause it to go underrecognized. Additionally, the time from symptom onset to MRI is variable among different patients, which is an inherent drawback related to the retrospective nature of this study. We could also not follow patients longitudinally to assess the reversibility of imaging findings over time. A prior case report in a patient with DPHL from carbon monoxide intoxication showed gradual resolution of restricted diffusion but persistence of the abnormal periventricular T2 signal abnormality which remained largely unchanged for over a year (Kim et al. 2002). Another case report documented incomplete resolution of the white matter abnormalities 1 year postoverdose (Shprecher et al. 2008), while separate reports showed improved but persistent abnormalities 6 months and 8 years after initial presentation, respectively (Molloy et al. 2006; Betts et al. 2012). Two-year clinical follow-up on another study, in patients with carbon monoxide-related DPHL, showed 75% recovery within 1 year (Choi 1983). Therefore, the use of the term “reversible” as in some of the prior descriptions of the disease might not be appropriate. Finally, DPHL has been shown to usually occur in older individuals. None of the patients in a large study of DPHL after carbon monoxide poisoning was less than 30 years of age (Choi 1983). Our patients were close to or above 60 years of age, with the exception of one patient who was 32 years old. Interestingly, this younger patient showed the most rapid clinical improvement of our cohort and was at his neurological baseline at discharge.

## Conclusion

The characteristics and distribution of imaging findings in DPHL can be striking on MRI. We have performed an exhaustive review of the literature on this entity and present our findings on its imaging aspects in great detail. In the appropriate clinical setting, bilateral and symmetric white matter signal abnormalities confined to the supratentorial white matter without gyral edema or enhancement, are highly suggestive of DPHL, which carries a relatively favorable prognosis compared to other acute toxic or metabolic causes of white matter injury.
